# Machine Learning for Predicting Long-Term Cardiovascular Outcomes in Kidney Transplant Recipients

**DOI:** 10.1016/j.jacadv.2025.102364

**Published:** 2025-11-18

**Authors:** William D. Park, Ava DeLonais-Parker, Barbara Okeke, Krista L. Lentine, Mina M. Benjamin, the SLU Transplant Rx research Group

**Affiliations:** a Saint Louis University School of Medicine, St. Louis, Missouri, USA; b Department of Internal Medicine, SSM Health-Saint Louis University Hospital, St. Louis, Missouri, USA; c Division of Nephrology, Department of Internal Medicine, SSM Health-Saint Louis University Hospital, St. Louis, Missouri, USA; d Division of Cardiology, Department of Internal Medicine, SSM Health-Saint Louis University Hospital, St. Louis, Missouri, USA

**Keywords:** echocardiography, kidney transplantation, predictive modeling, risk stratification

## Abstract

**Background:**

Cardiovascular disease is the leading cause of morbidity and mortality in kidney transplant recipients (KTRs). Conventional risk assessment tools often underperform in this population due to their unique cardiovascular milieu shaped by end-stage kidney disease and immunosuppression.

**Objectives:**

This study aimed to evaluate the performance of machine learning (ML) models in predicting major adverse cardiovascular events in KTRs.

**Methods:**

We analyzed a data set of 518 adult KTRs from a single center (2015-2024) incorporating 1,014 demographic, clinical, laboratory, and imaging parameters in addition to ischemic work-up and revascularization details. After preprocessing, 44 features were used to train and validate ML models using AutoGluon with 5-fold cross-validation and permutation-based feature selection as well as domain knowledge. SHAP was used for model interpretation. The best performing ML model was compared to multivariable logistic regression in addition to a historical model from prior literature, that is, Soveri risk score.

**Results:**

Over a mean follow-up of 5.3 ± 2.3 years, 102 patients experienced major adverse cardiovascular events (mean age 52.9 ± 13.2 years; 56.9% male). The CatBoost model achieved the highest area under the receiver-operating characteristic curve (AUROC) (0.72; 95% CI: 0.607-0.811) and area under the precision-recall curve (0.36; 95% CI: 0.230-0.540), greater than the logistic regression (AUROC = 0.67; 95% CI: 0.559-0.772, area under the precision-recall curve = 0.31; 95% CI: 0.228-0.553). The differences were not statistically significant. Both models had significantly higher AUROC than the Soveri risk score. Top predictive variables included coronary artery disease, left atrial reservoir strain, dialysis duration, and left ventricular end-diastolic volume index.

**Conclusions:**

ML models are feasible for predicting cardiovascular events in KTRs.

Cardiovascular disease is the leading cause of morbidity and mortality in kidney transplant recipients (KTRs).^1^, ^2^, ^3^ In addition to traditional cardiovascular risk factors, this population faces unique risks related to end-stage kidney disease (ESKD) and immunosuppressive therapy.^4^^,^^5^ Current risk assessment tools, including traditional scoring systems, imaging modalities, and even invasive angiography, show limited accuracy in predicting cardiovascular risk as demonstrated in clinical trials.^6^^,^^7^ This gap in risk stratification contributes to suboptimal management and potentially preventable adverse outcomes.

Prior studies have used machine learning (ML) for predicting graft survival and patient mortality following kidney transplant (KT) with moderate success, demonstrating the potential utility of ML approaches.^8^ Previous studies have identified predictors, such as age, hypertension, and diabetes.^9^ Because of the unconventional cardiovascular milieu KTRs live through, ML could identify cardiovascular risk factors in this population, that conventional statistical models may overlook. Given the subpar performance of standard risk calculators in KTRs, cardiovascular risk calculators specific to KTRs have been developed. However, the robustness of the database used for developing the ML models has varied significantly.^10^, ^11^, ^12^ We aimed to compare the performance of ML to traditional multivariable logistic regression models in predicting long-term major adverse cardiovascular events (MACE) in KTRs, using a robust, granular database.

## Methods

Study design: After obtaining approval from our Institutional Review Board (protocol #34091), we built a retrospective database of adult (age ≥18 years) KTRs at Saint Louis University Hospital between January 2015 and January 2024. We excluded patients who did not have follow-up at our health care system. Patient charts were reviewed for 1,014 pretransplant variables including demographic, clinical, laboratory values, medications as well as pretransplant cardiac ischemic evaluation and revascularization data. Supplemental Appendix 2 contains a dictionary of the variables recorded and their definitions. In addition, strain measurements were performed for the right ventricle (RV), left atrium (LA) as well as left ventricle (LV) using TomTec software (TomTec Imaging Systems GmbH). RV strain was measured at the basal, mid, and apical free wall and averaged. LA strain was measured in the 2- and 4-chamber views then averaged. Endocardial borders were manually traced at end-systole in standard apical 2-, 3-, and 4-chamber views, and automated speckle-tracking was used to generate longitudinal strain curves for each segment. Global longitudinal strain was calculated as the average peak systolic strain from all segments of the LV. The principal investigator (M.M.B.) has completed a year of fellowship training in advanced cardiac imaging. Investigators were trained by the principal investigator on performing strain measurements and were required to achieve an interobserver agreement rate of at least 80% with the principal investigator on a random sample of 15 patients before participating in the study. The primary study endpoint was defined as any MACE post-KT, defined as acute coronary syndrome, stroke, hospitalization for heart failure, need for revascularization, and sustained ventricular arrhythmias. For illustration in this manuscript, patients were divided into two groups based on whether they developed a MACE event (MACE vs no MACE). Continuous variables were compared between MACE vs no-MACE groups with a *t*-test or Mann-Whitney *U* test (depending on distribution), and categorical variables with a chi-square test or Fisher exact test. All CIs are reported at the 95% level.

Data preprocessing and feature engineering: To ensure transparent and standardized reporting of our ML model, we followed the TRIPOD-AI (Transparent Reporting of a multivariable prediction model for Individual Prognosis or Diagnosis–Artificial Intelligence extension) checklist. This framework guided the documentation of model development, feature selection, validation procedures, performance metrics, and handling of potential biases, supporting reproducibility and interpretability of our findings. Initially, 1,014 features were present in the data set. Patients with missing demographic information or values outside of plausible clinical range were removed. Features with more than 30% missing values and low variance across the whole data set were also removed, resulting in 66 features. Additional columns of data with aggregated features were added to enhance interpretability and variables with low variance were removed. After data cleaning, the data consisted of 58 features. For ensemble model training and validation, AutoGluon-Tabular classifier was used (version 1.1.1).^13^ The data were randomly split 70% for training and 30% for testing with approximately equal positive event rate. Categorical features were one-hot-encoded. Data imputation was completed via MissForest, which is a random forest imputation method. The same training data were used for both preprocessing and model training.

**Figure 1:**
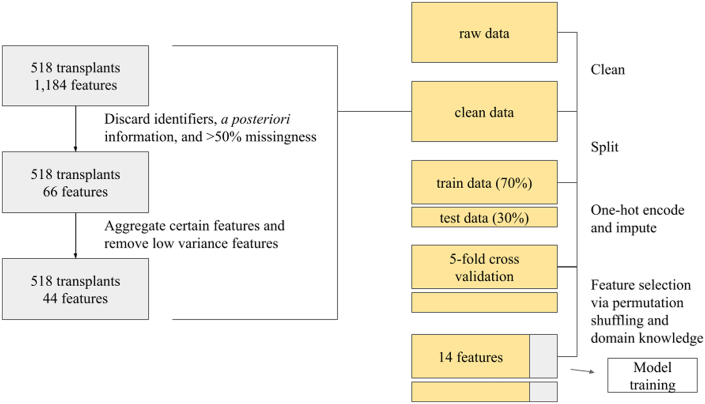
**End-to-End Data Processing Pipeline** The data cleaning process from raw data to processed data is detailed on the left. Post-transplant evaluation data and features with more than 50% missingness, a posteriori *information*, and identifiers were removed. Certain features were aggregated to enhance interpretability and low variance features were removed. Five-fold cross-validation using the LightGBM model was performed during feature selection independently for each fold as well as in the final model training.

Feature selection and model development: Permutation importance was calculated through five-fold cross-validation using 44 features. The LightGBM model was used for each fold to calculate permutation importance and those with frequency of at least 3 or greater across all the folds were included in the final features. Features that were added based on domain expertise include age at KT, hemoglobin A1C, dialysis duration, Black race, diastolic LV posterior wall thickness, and total cholesterol. Fourteen final features were selected. Figure 1 illustrates the data cleaning process. Feature importance evaluation was completed using AutoGluon’s “feature_importance” attribute. Models were trained using five-fold cross-validation with bootstrap aggregation, and model performance was assessed using the area under the receiver-operating characteristic curve (AUROC) as well as the area under the precision-recall curve (AUPRC). Models used include LightGBM, CatBoost, XGBoost, random forest, extra trees, k-nearest neighbors, PyTorch network, and fastai v1 neural network. Additionally, stack ensembling was used to leverage the predictions of all the models and generate a weighted ensemble. A historical risk calculator using 8 different features from Soveri et al was used as well as a reference.^12^ Using the formula listed in Soveri et al, probabilities were calculated using the 8 features (age, post-KT creatinine, low density lipoprotein, former smoker, current smoker, diabetes mellitus, number of transplants, and coronary artery disease [CAD]). The best performing ML model was then compared to a multivariable logistic regression model computed in R (version 4.3.1) using the same exact features as the ML models for direct comparison. Multicollinearity was assessed using variance inflation factors. The Soveri risk score was calculated too as a historical benchmark. The final ML models were interpreted using SHapley Additive exPlanations (SHAP) to provide clinical insight. Differences in AUROC and AUPRC between models were assessed using a bootstrap approach (eg, bootstrapping the prediction sets to derive CIs and *P* values for AUPRC differences). AUPRC was designated as the primary metric for model selection given the class imbalance. To evaluate predicted and observed outcomes, calibration curves were generated for the top ML, logistic regression, and Soveri models. Decision curve analysis was also performed to evaluate the potential clinical utility of these models. All statistical tests were 2-sided, with *P* < 0.05 considered significant.

## Results

After excluding patients without follow-up at our institution, 518 KTRs were included in the study (mean age 52.9 ± 13.2 years; 56.9% male). Over a mean follow-up period of 5.3 ± 2.3 years following KT, 102 patients experienced at least 1 MACE. Baseline demographics and clinical characteristics for the entire cohort and both subgroups are presented in Table 1. Echocardiographic characteristics are summarized in Table 2. A breakdown of events is listed in Table 3. Black KTRs experienced a significantly higher incidence of MACE compared to other ethnicities (60.8% vs 44.2%). Patients who experienced MACE were significantly older (56.1 ± 12.2 vs 52.2 ± 13.3 years) and had a higher prevalence of CAD (48.0% vs 24.3%). A significantly greater proportion of MACE patients (70.6% vs 57.2%) were on hemodialysis rather than peritoneal dialysis or no dialysis before KT. Additionally, among those on dialysis, patients in the MACE group had a longer duration of dialysis (55.0 vs 39.8 months). Hemoglobin A1c was also significantly higher in the MACE group (5.9 ± 1.2 vs 5.5 ± 0.9). Notably, LV ejection fraction did not differ significantly between the groups. However, LA strain parameters—reservoir (26.8 ± 10.4 vs 31.4 ± 11.0), contractile (13.8 ± 5.7 vs 15.5 ± 8.1), and conduit (13.4 ± 6.6 vs 16.9 ± 7.6)—were significantly lower in the MACE group. Right ventricular (RV) free wall strain (18.9 ± 6.7 vs 21.1 ± 8.8) and LV global longitudinal strain (14.5 ± 3.4 vs 15.8 ± 4.2) were also significantly lower in patients who experienced MACE.

**Table 1:** Baseline Clinical Characteristics

	Total (N = 518)	MACE (n = 102)	No MACE (n = 416)	*P* Value
Male (%)	295 (56.9)	60 (58.8)	235 (56.5)	0.753
Age, mean ± SD (y)	52.9 ± 13.2	56.1 ± 12.2	52.2 ± 13.3	**0.005**
Race				
White (%)	242 (46.7)	39 (38.2)	203 (48.8)	0.071
Black (%)	246 (47.5)	62 (60.8)	184 (44.2)	**0.004**
Other (%)	30 (5.8)	1 (1.0)	29 (7.0)	**0.037**
Body mass index (kg/m^2^), mean ± SD	29.6 ± 5.7	29.7 ± 5.5	29.5 ± 5.7	0.778
Tobacco use (%)	162 (31.3)	31 (30.4)	131 (31.5)	0.924
Comorbidities				
Hypertension (%)	463 (89.3)	91 (89.2)	372 (89.4)	1.000
Obstructive sleep apnea (%)	152 (29.3)	34 (33.3)	118 (28.4)	0.419
Type 2 diabetes mellitus (%)	169 (32.6)	41 (40.2)	128 (30.8)	0.075
Coronary artery disease (%)	150 (29.0)	49 (48.0)	101 (24.3)	**<0.001**
Hemodialysis (%)	310 (59.8)	72 (70.6)	238 (57.2)	**0.018**
Peritoneal dialysis (%)	142 (27.4)	24 (23.5)	118 (28.4)	0.391
Dialysis duration, mean ± SD (mo)	42.7 ± 37.5	55.0 ± 42.4	39.8 ± 35.7	**0.002**
Coronary artery disease screening modality				
Invasive coronary angiography (%)	248 (47.9)	65 (63.7)	183 (44.0)	**<0.001**
Nuclear stress test (%)	147 (28.4)	33 (32.4)	114 (27.4)	0.384
Stress echocardiography (%)	350 (67.6)	66 (64.7)	284 (68.3)	0.568
Medications				
Aspirin (%)	232 (44.8)	62 (60.8)	170 (40.9)	**<0.001**
Clopidogrel (%)	24 (4.6)	8 (7.8)	16 (3.8)	0.111
Any anticoagulation (%)	30 (5.8)	7 (6.9)	27 (6.5)	0.98
Beta-blocker (%)	320 (61.8)	65 (63.7)	255 (61.3)	0.735
ACE inhibitor or ARB (%)	187 (36.1)	33 (32.4)	154 (37.0)	0.445
Calcium-channel blocker (%)	263 (50.8)	47 (46.1)	216 (51.9)	0.343
Diuretic (%)	140 (27.0)	22 (21.6)	118 (28.4)	0.207
Statin (%)	332 (64.1)	77 (75.5)	255 (61.3)	**0.010**
Hydralazine (%)	93 (18.0)	19 (18.6)	74 (17.8)	0.981
Nitrites (%)	25 (4.8)	9 (8.8)	16 (3.8)	0.078
Laboratory values				
BNP, mean ± SD	2,432.5 ± 6,165.3	1,355.2 ± 1,992.7	2,755.6 ± 6,922.8	0.092
Hemoglobin, mean ± SD	10.7 ± 1.9	10.6 ± 1.9	10.7 ± 1.9	0.537
Hemoglobin A1C, mean ± SD	5.6 ± 1.0	5.9 ± 1.2	5.5 ± 0.9	**0.012**
Troponin I, mean ± SD	0.4 ± 3.3	0.2 ± 0.3	0.1 ± 0.3	0.636

ACE = angiotensin-converting enzyme; ARB = angiotensin receptor blocker; BNP = B-type natriuretic peptide; MACE = major adverse cardiovascular event.

Significant *P* values are in **bold**.

**Table 2:** Baseline Echocardiographic Characteristics

	Total (N = 518)	MACE (n = 102)	No MACE (n = 416)	*P* Value
LVEF, mean ± SD	62.7 ± 8.3	61.2 ± 8.4	63.1 ± 8.3	0.069
TAPSE, mean ± SD	6.8 ± 8.7	7.0 ± 9.5	6.7 ± 8.6	0.807
IVSd, mean ± SD	1.3 ± 0.4	1.3 ± 0.4	1.3 ± 0.4	0.523
LVPWd, mean ± SD	1.2 ± 0.3	1.2 ± 0.3	1.2 ± 0.3	0.753
RVSP, mean ± SD	34.3 ± 9.3	35.7 ± 7.5	34.0 ± 9.7	0.220
TR peak gradient, mean ± SD	26.2 ± 13.5	29.3 ± 24.8	24.5 ± 9.1	0.203
LVEDVI, mean ± SD	63.7 ± 23.8	64.7 ± 25.0	63.5 ± 23.7	0.781
LV mass, mean ± SD	127.7 ± 77.8	133.2 ± 71.6	126.6 ± 79.0	0.506
LVESVI, mean ± SD	23.2 ± 12.2	23.4 ± 11.7	23.2 ± 12.3	0.916
Pericardial effusion (%)	97 (18.7)	19 (18.6)	78 (18.8)	0.990
RA area, mean ± SD	16.1 ± 4.4	16.5 ± 4.6	16.0 ± 4.4	0.488
LA volume, mean ± SD	81.4 ± 31.2	69.8 ± 39.4	65.3 ± 28.9	0.294
LA max area, mean ± SD	41.6 ± 15.3	39.5 ± 9.4	42.0 ± 16.2	0.095
LA max length, mean ± SD	11.3 ± 3.3	10.9 ± 1.3	11.4 ± 3.6	0.069
LA reservoir strain, mean ± SD	30.4 ± 11.0	26.8 ± 10.4	31.4 ± 11.0	**0.037**
LA contractile strain, mean ± SD	15.1 ± 7.7	13.8 ± 5.7	15.5 ± 8.1	**<0.001**
LA conduit strain, mean ± SD	16.2 ± 7.6	13.4 ± 6.6	16.9 ± 7.6	**<0.001**
RV free wall strain, mean ± SD	20.6 ± 8.4	18.9 ± 6.7	21.1 ± 8.8	**0.016**
LV global longitudinal strain, mean ± SD	15.5 ± 4.0	14.5 ± 3.4	15.8 ± 4.2	**0.005**
Mitral valve regurgitation				
None or trace (%)	316 (61.0)	55 (53.9)	261 (62.7)	0.128
Mild (%)	145 (28.0)	32 (31.4)	113 (27.2)	0.468
Moderate (%)	19 (3.7)	6 (5.9)	13 (3.1)	0.234
Aortic valve regurgitation				
None or trace (%)	411 (79.3)	69 (67.6)	342 (82.2)	**0.002**
Mild (%)	60 (11.6)	21 (20.6)	39 (9.4)	**0.003**
Moderate (%)	6 (1.2)	2 (2.0)	4 (1.0)	0.337
Aortic valve stenosis				
None or trace (%)	451 (87.1)	84 (82.4)	367 (88.2)	0.156
Mild (%)	15 (2.9)	4 (3.9)	11 (2.6)	0.510
Moderate (%)	4 (0.8)	1 (1.0)	3 (0.7)	0.585

IVSd = interventricular septum thickness in diastole; LA = left atrial; LV = left ventricular; LVEDVI = left ventricular end-diastolic volume index; LVEF = left ventricular ejection fraction; LVESVI = left ventricular end-systolic volume index; LVPWd = left = ventricular posterior wall thickness in diastole; RA = right atrial; RV = right ventricular; RVSP = right ventricular systolic pressure; TAPSE = tricuspid annular plane systolic excursion; TR = tricuspid regurgitation; other abbreviation as in Table 1.

Significant *P* values are in **bold**.

**Table 3:** Patient Outcomes

All-cause mortality	38 (37.3)
Cardiovascular death	17 (16.7)
Nonfatal myocardial infarction	24 (23.5)
Cerebrovascular accident	21 (20.6)
Unstable angina	12 (11.8)
Heart failure	43 (42.2)
Ventricular arrhythmia	9 (8.8)
Need for coronary revascularization	15 (14.7)

Values are n (%).

**Figure 2:**
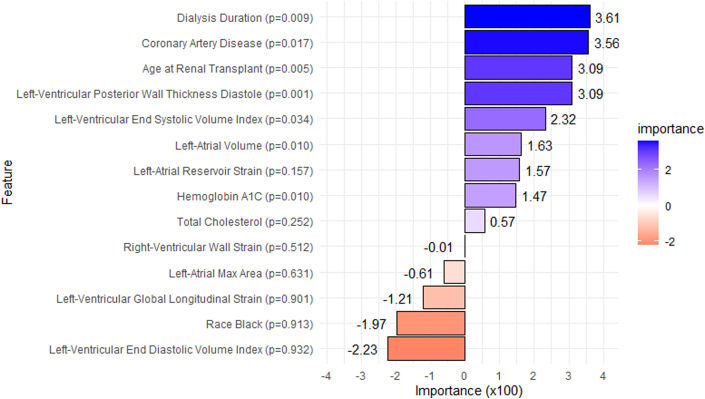
**Feature Importance Based on Permutation Shuffling** Importance values were upscaled by a factor of 100 to improve readability. Positive importance denotes significant impact in model prediction, whereas negative importance denotes hindrance in model prediction. Low importance based on permutation shuffling may suggest a nonlinear relationship or interaction-based effects. *P* values are listed next to each feature. Most important features are on top.

**Figure 3:**
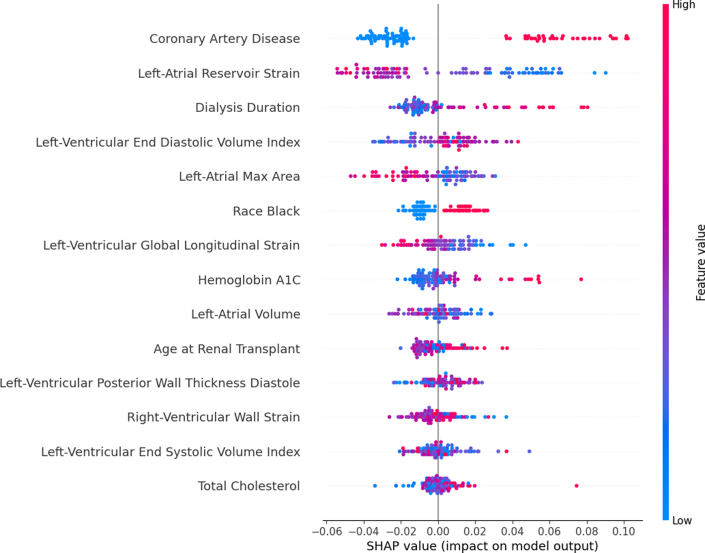
**Beeswarm Plot of SHAP Values for the CatBoost_r128 Model** Each point represents a single feature data point. The color gradient represents whether the feature value itself was low or high, whereas the magnitude along the x-axis determines the SHAP value impact on model output. SHAP = SHapley Additive exPlanations.

**Figure 4:**
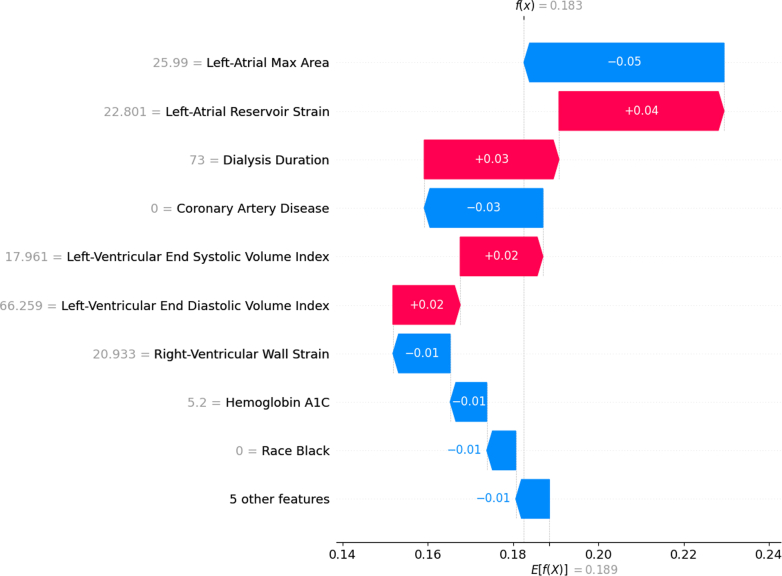
**Waterfall Plot of the CatBoost_r128 Model** Red bars show that the feature pushes toward positive predictions of MACE, whereas blue bars show that the feature pushes toward negative predictions of MACE. Magnitude of bar denotes relative impact toward final prediction. E[f(x)] represents the expected prediction based on all feature averages, whereas f(x) represents the final, tailored prediction. Most important features are on top. Actual feature values for this particular prediction are listed in gray to the left of the feature name. MACE = major adverse cardiovascular event.

Permutation importance scores (scaled by 100) and corresponding *P* values are shown in Figure 2. Dialysis duration had the highest importance score (3.61). Other high-importance features included CAD (3.56), age (3.09), diastolic LV posterior wall thickness (3.09), and LV end-systolic volume index (2.32). For clinical interpretability, Figure 3 illustrates SHAP value distributions and feature impacts for the best-performing ML model, a CatBoost-based ensemble. The top 5 predictive features in this model were CAD, LA reservoir strain, dialysis duration, LV end-diastolic volume index, and LA max area. Figure 4 demonstrates how individual feature values influenced predictions in a representative case. Models were evaluated using F1 score, recall, precision, AUROC, and AUPRC as detailed in Supplemental Appendix 3. Tables 4 and 5 summarize the top 5 models based on these metrics. Notably, the CatBoost_r128 model achieved the highest AUPRC at 0.363 (95% CI: 0.230-0.540), with relatively balanced recall at 0.400 (95% CI: 0.269-0.575) and precision at 0.645 (95% CI: 0.481-0.919). The AUROC for this model was 0.716 (95% CI: 0.607-0.811).

**Table 4:** Summary of the Top Five Machine Learning Models for Predicting Major Adverse Cardiovascular Events Post-Kidney Transplant

Model	F1 Score (CI)	Recall (CI)	Precision (CI)
CatBoost_r128	0.494 (0.383-0.622)	0.400 (0.269-0.575)	0.645 (0.481-0.919)
CatBoost_r180	0.495 (0.387-0.615)	0.364 (0.273-0.545)	0.774 (0.480-0.913)
NeuralNetTorch	0.432 (0.354-0.588)	0.372 (0.233-0.606)	0.516 (0.400-0.941)
RandomForest	0.433 (0.325-0.590)	0.448 (0.220-0.728)	0.419 (0.310-0.921)
RandomForestGini	0.433 (0.325-0.590)	0.448 (0.220-0.728)	0.419 (0.310-0.921)

**Table 5:** Performance Metrics of the Top Five Machine Learning Models for Predicting Major Adverse Cardiovascular Events Post-Kidney Transplant

Model	AUROC (CI)	AUPRC (CI)
CatBoost_r128	0.716 (0.607-0.811)	0.363 (0.230-0.549)
CatBoost_r180	0.718 (0.612-0.814)	0.361 (0.233-0.549)
NeuralNetTorch	0.692 (0.588-0.788)	0.367 (0.228-0.528)
RandomForest	0.653 (0.538-0.761)	0.356 (0.210-0.550)
RandomForestGini	0.653 (0.538-0.761)	0.356 (0.210-0.550)

AUROC = area under the receiver-operating characteristic curve; AUPRC = area under the precision-recall curve.

**Table 6:** Adjusted Logistic Regression Analysis for Major Adverse Cardiovascular Events in Kidney Transplantation Recipients

	OR	95% CI	*P*-Value
Age	1.01	0.99–1.04	0.327
Black race	2.02	1.12–3.73	**0.021**
Coronary artery disease	2.80	1.51–5.24	**0.001**
Dialysis duration	1.01	1.00–1.01	0.202
Hemoglobin A1C	1.12	0.86–1.46	0.390
Total cholesterol	1.00	1.00–1.01	0.158
Left ventricular posterior wall thickness diastole	0.92	0.29–2.72	0.886
Left ventricular end systolic volume index	1.00	0.95–1.04	0.880
Left ventricular end-diastolic volume index	1.00	0.98–1.03	0.880
Left ventricular global longitudinal strain	0.99	0.88–1.10	0.802
Left atrial max area	0.96	0.90–1.01	0.201
Left atrial volume	1.00	0.99–1.02	0.527
Left atrial reservoir strain	0.95	0.91–0.99	**0.018**
Right ventricular wall strain	1.01	0.96–1.06	0.805

Significant *P* values are in **bold**.

**Figure 5:**
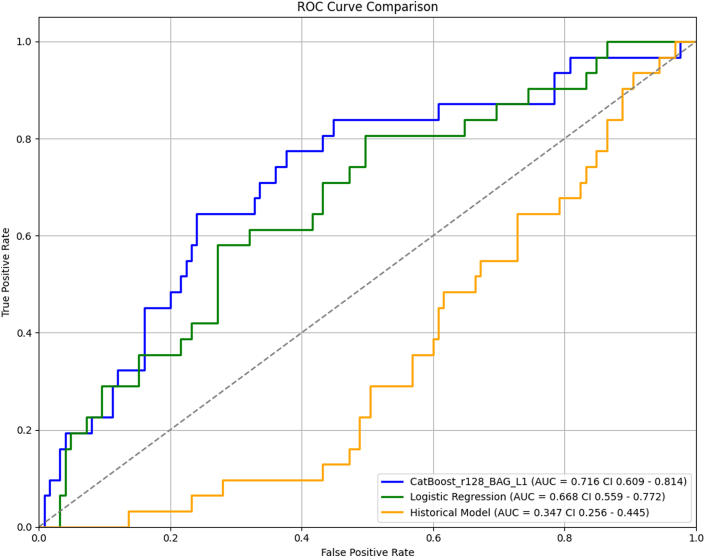
Receiver Operating Characteristic Curve comparison between the 3 models: logistic regression model (green), the CatBoost_r128 model (blue), and the soveri risk score. AUC = area under the curve.

**Figure 6:**
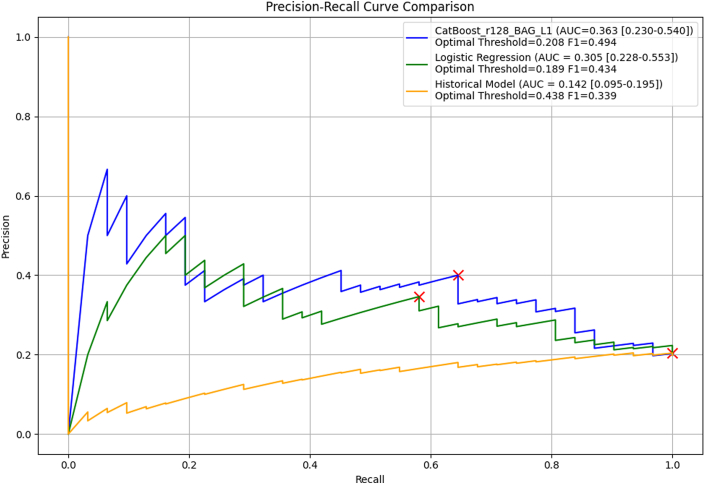
Precision Recall Curve comparison between the 3 models: logistic regression model (green), the CatBoost_r128 model (blue), and the soveri risk score.

**Figure 7:**
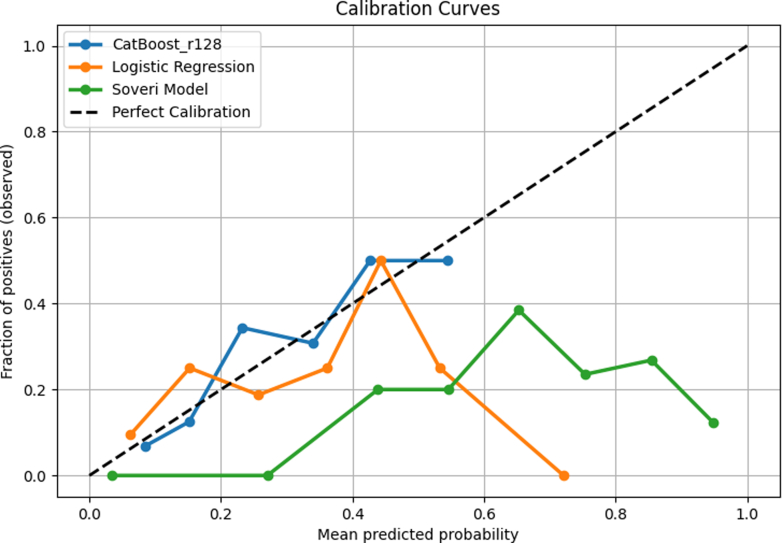
**Calibration Curve Comparison** The CatBoost_r128, logistic regression, and Soveri models are compared. The dashed diagonal line represents perfect calibration where predicted risk is equal to observed risk. Points above the line suggest underestimation, whereas points below suggest overestimation.

**Figure 8:**
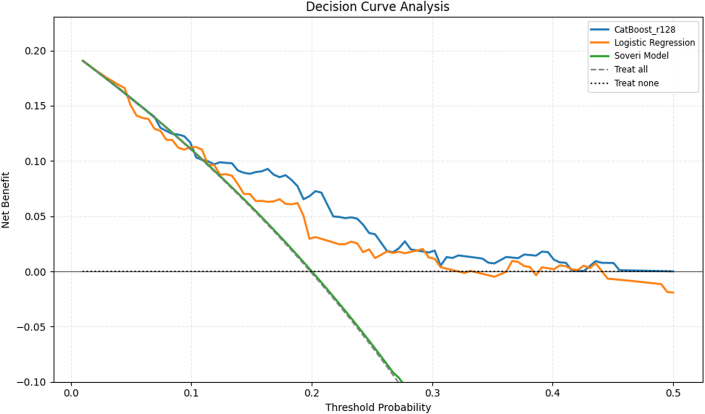
**Decision Curve Analysis** The CatBoost_r128, logistic regression, and Soveri models are compared.

In the multivariable regression model (Table 6), unstable angina (OR: 6.15; 95% CI: 1.70-25.41; *P* = 0.007), CAD (OR: 2.08; 95% CI: 1.10-3.94; *P* = 0.024), and LA conduit strain (OR: 0.93; 95% CI: 0.88-0.97; *P* = 0.003) were independently associated with MACE. Variance inflation factors ranged from 1.1 to 1.8, confirming no problematic multicollinearity. Figure 5 compares receiver-operating characteristic curves between the models. The ML model (AUROC = 0.716; 95% CI: 0.607-0.811) had a greater area than both the logistic regression model (AUROC = 0.668; 95% CI: 0.559-0.772) and the Soveri risk score (AUROC = 0.347; 95% CI: 0.256-0.445), but that difference was not statistically significant (*P* = 0.224) for the former but was significant for the latter (*P* < 0.001). Logistic regression model compared to the Soveri risk score had a significant *P* value of <0.001. Figure 6 compares the precision-recall curves between the 3 models. Similarly, the ML model (AUPRC = 0.363; 95% CI: 0.230-0.540) had a greater area compared to the logistic regression model (AUPRC = 0.305; 95% CI: 0.228-0.553) as well as the Soveri risk score (AUPRC = 0.142; 95% CI: 0.095-0.195). None of the AUPRC differences reached significance. The P values for the difference in AUROC and AUPRC for each two of the 3 models are listed in Table 7. Figure 7 shows the calibration curves for the CatBoost_r128, logistic regression, and Soveri models. The CatBoost_r128 model is closest to the perfect calibration line, suggesting the best prediction accuracy. Additionally, the CatBoost_r128 model had the lowest Brier score, that is, 0.147. Figure 8 shows the decision curve analysis for the CatBoost_r128, logistic regression, and Soveri models. Visually, the CatBoost_r128 model provided greater net benefit improvement compared to the logistic regression and Soveri models across nearly all thresholds (Central Illustration).

**Table 7:** P-Value Comparisons of AUPRC and AUROC Between the Machine Learning Model, Logistic Regression, and Soveri Risk Score

Model 1	Model 2	AUROC *P*-Value
CatBoost_r128	Logistic regression	0.224
CatBoost_r128	Soveri risk score	<0.001
Logistic regression	Soveri risk score	<0.001

Model 1	Model 2	AUPRC *P* Value
CatBoost_r128	Logistic regression	0.948
CatBoost_r128	Soveri risk score	0.930
Logistic regression	Soveri risk score	0.984

Abbreviations as in Table 5.

**Central Illustration:**
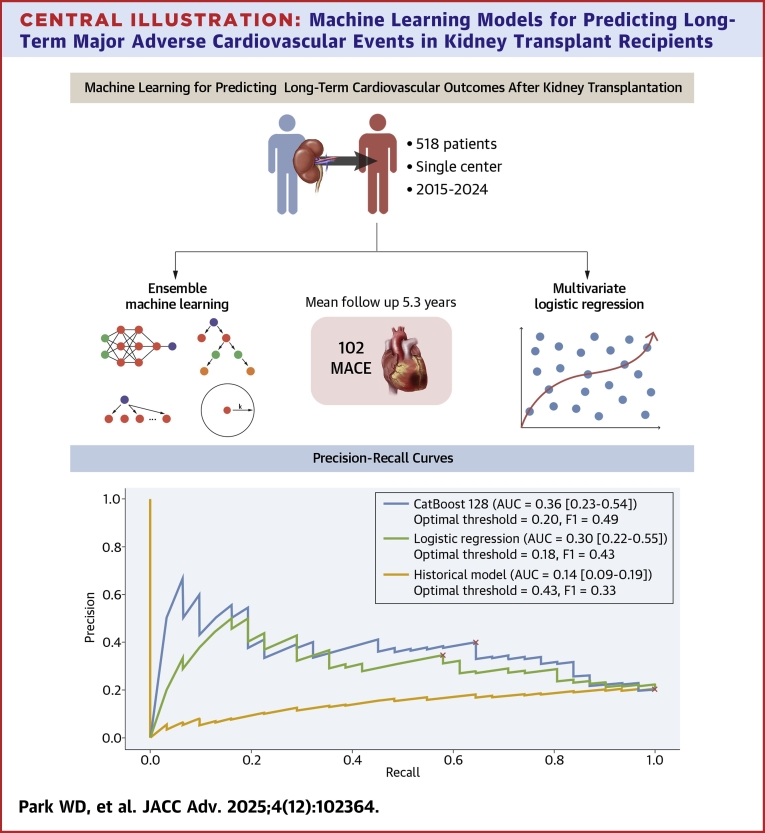
**Machine Learning Models for Predicting Long-Term Major Adverse Cardiovascular Events in Kidney Transplant Recipients** Abbreviation as in Figure 4.

## Discussion

In this retrospective study of KTRs, we aimed to prove the feasibility of developing ML models for predicting MACE following KT, with reasonable predictive performance. This robust analysis of a comprehensive clinical data set yielded several key findings. 1) ML models consistently tended to have a better performance compared to traditional logistic regression in predicting MACE after KT, though without a statistically significant difference. 2) The key predictors of MACE in our study included some previously recognized demographic and clinical variables (Black race, diabetes, unstable angina, and dialysis duration) as well as some of the echocardiographic features, particularly LA and RV strain, underscoring the importance of subclinical cardiac remodeling.

In our study, consistent with prior studies, Black race remained with a strong association with poor cardiac outcomes. Black race patients with ESKD get initiated on dialysis later than other races and have lower erythropoietin use.^14^, ^15^, ^16^ This racial disparity could not be explained by differences in renal function, suggesting that additional socioeconomic factors are leading to these differences.^17^ In our analysis, having CAD specially patients who experienced angina was associated with the outcome. This has been shown in several prior studies, underscoring the persistent need for better risk mitigation strategies in those patients.^18^, ^19^, ^20^, ^21^, ^22^ The other traditional risk factors identified in our study like age, increased dialysis duration, and uncontrolled diabetes are also consistent with prior literature.^23^ A retrospective study conducted by Ardehali et al demonstrated that KTRs with MACE were significantly older, more likely to have CAD, cardiomyopathy, smoking history, and diabetes.^24^ Another retrospective study involving 2,187 KTRs found that diabetes leading to diabetic nephropathy, longer duration on dialysis, older age, higher body mass index, and prior cardiac events were significant predictors of MACE and all-cause mortality.^25^

In our models, LA max area, volume as well as LA strain components were associated with MACE. We kept both LA area (single-plane planimetry) and volume (biplane Simpson) in the model as they may capture different measurement aspects and missingness profiles; tree-based ML (CatBoost) is robust to correlated inputs, so predictive performance was not harmed. LA enlargement is a common pathology in ESKD due to LV dysfunction secondary to hypertension and volume overload. Additionally, many patients on dialysis have arteriovenous fistulas, which can contribute to increases in cardiac preload.^26^ Prior studies have demonstrated that LA strain components are associated with MACE in several other cardiac conditions.^27^, ^28^, ^29^ Other echocardiographic variables that had a higher importance in the prediction models were LV end diastolic and systolic volume indices.^30^^,^^31^ These parameters reflect elevated filling pressures and adverse ventricular remodeling, which are common in ESKD and may persist post-transplant due to long-standing hypertension, volume overload, and arteriovenous fistula flow. Prior studies have shown that LV dilation is associated with worse cardiovascular outcomes and all-cause mortality in both dialysis and KT populations.^30^^,^^31^ LV strain as well has been previously shown to associate with adverse outcomes in a smaller study.^32^ RV strain also emerged with a strong association with the outcome. Abnormal RV strain denotes RV dysfunction that is likely a sequela of volume overload and pulmonary hypertension that are highly prevalent in ESKD patients, in addition to uremic myocardial remodeling. Several studies have demonstrated that even after transplantation, RV abnormalities may persist, reflecting residual, irreversible myocardial injury.^33^^,^^34^ Pulmonary hypertension was not included in our final model but have been reported in multiple studies to associate with the adverse outcomes.^34^, ^35^, ^36^ The lack of a significant association in our study is likely because the vast majority of pulmonary artery pressure estimation at our center relied on echocardiography, rather than the gold standard of right heart catheterization.^34^^,^^35^

Several variables previously associated with adverse outcomes after KT—such as LV ejection fraction, body mass index, and smoking history—did not emerge as significant predictors in our study. One possible explanation is that our cohort had relatively preserved LV ejection fraction overall, limiting its discriminatory power. Smoking status may have been underreported or lacked sufficient variability in this retrospective data set, reducing their predictive contribution.

Notably, the older risk calculator by Soveri et al performed poorly in our cohort (AUROC ∼0.35), underscoring the need for updated models tailored to contemporary KTR populations. In our study, ML models tended to outperform traditional multivariable logistic regression in predicting MACE. This is likely attributable to the ML model's ability to capture complex, nonlinear relationships and interactions between a large number of input variables. In contrast, logistic regression relies on linear assumptions which may have limited its capacity to account for subtler patterns in the data. Moreover, the ensemble nature of the ML model and the use of automated feature selection and hyperparameter tuning likely contributed to its enhanced predictive performance. Additionally, while ML models demonstrated higher predictive performance, they also come with challenges in interpretability and clinical integration. Overall, there seems to be ample room for improving risk prediction in KT candidates. Future studies should investigate whether integrating images from the cardiac work-up (radiomics) could improve the model’s ability to predict events. Future ML models should also be externally validated to improve generalizability.

### Study Limitations

Our study has several limitations to consider. First, the retrospective design inherently limits causal inference and is subject to documentation bias. Despite rigorous preprocessing, missing data remained a challenge, potentially introducing selection bias. The analysis was limited to a single urban academic medical center, which may restrict the generalizability of the findings to other institutions with different patient populations, clinical protocols, or health care systems. While SHAP values provided insight into model interpretability, the inherent complexity of ensemble ML models such as LightGBM, CatBoost, and XGBoost may limit their transparency and clinical adoption, particularly when decision-making must be explained to stakeholders. Additionally, the use of permutation-based feature importance may fail to detect nonlinear interactions or synergistic effects between variables, possibly overlooking meaningful predictors. Our approach of adding domain-expertise features was meant to mitigate that limitation. The study cohort was relatively small, which increases the risk of model overfitting despite the use of a five-fold cross-validation and a 70/30 train-test split. Moreover, class imbalance (MACE event rate of 19.7%) may have affected model calibration and contributed to modest F1 scores. Alternative approaches such as synthetic minority over-sampling technique or cost-sensitive learning were not applied and may have improved performance. Finally, patients without post-transplant follow-up at our institution were excluded, which could introduce attrition and selection bias. This subgroup may have had different clinical outcomes, further limiting external validity.

## Conclusions

ML models integrating pretransplant clinical and echocardiographic data can potentially improve individualized cardiovascular risk prediction in KTRs. However, external validation is essential before clinical implementation.

Perspectives **COMPETENCY IN MEDICAL KNOWLEDGE:** ML models may be able to outperform traditional risk score calculators for predicting long-term cardiovascular events in KTRs.

**COMPETENCY IN PATIENT CARE AND PROCEDURAL SKILLS:** Identifying patients with higher risk using ML with greater accuracy may help optimize both pre- and post-transplant care.

**TRANSLATIONAL OUTLOOK 1:** ML models can be incorporated into KT evaluation offering refined risk prediction to guide clinical decision-making and initiate early preventive strategies for patients who are at higher risk.

**TRANSLATIONAL OUTLOOK 2:** Integrating images from cardiac radiomics could improve the model’s ability to predict future cardiovascular outcomes.

## Funding support and author disclosures

This study was supported by the 10.13039/100011620Saint Louis University Internal Medicine Seed Grant. Dr Lentine is supported by the Mid-America Transplant/Jane A. Beckman Endowed Chair in Transplantation. Outside of this work, Dr Lentine has received consulting fees from CareDx and speaker honoraria from 10.13039/100004339Sanofi. All other authors have reported that they have no relationships relevant to the contents of this paper to disclose.
