# Supplementary material for: Machine Learning for Predicting Long-Term Cardiovascular Outcomes in Kidney Transplant Recipients
Source: JACC Adv. 2025 Nov 18;4(12):102364. doi: 10.1016/j.jacadv.2025.102364 (PMC12666815; doi:10.1016/j.jacadv.2025.102364)
Supplement: Supplemental Material [file mmc1.docx]

**Supplemental Appendix 1:** Acknowledgements

TNC Research Group members who assisted in data collection:

Saint Louis University:

Claire Elah Doua

Mark Schnitzler, PhD

Saint Louis University School of Medicine:

Ananta Sriram

Anna Bower

Cayden Lawrence

Emma Li

Gavin Christy

Hannah Wiseman

James Roe

Julia Lieu

Khang Nguyen

Leighton Hope

Michael Kanan

Ravneet Nagra

Samantha Harrington

Sophia Heuer

Spencer Hobbs

Taylor Coffman

Yanqing Lyu

Zalan Shah

SSM Saint Louis University Hospital:

Yasar Caliskan, MD

Chien-Jung Lin, MD

Venkata Peddada, DO

Eric Stein, MD

Mata Andres, MD

Shannon Ejiofor, DO

**Supplemental Appendix 2:** Data dictionary

Kidney Transplant Evaluation Form

| Field Label | Meaning | Data Type | Choices, Calculations, OR Slider Labels |
| --- | --- | --- | --- |
| AGE | Patient's age in years at time of transplant evaluation. | Numerical |  |
| RACE | Self-identified racial category of the patient. | Categorical | 1 Black/African American  2 American/Indian  3. Asian   4 Hispanic Latino  5 Native Hawaiian  6 White  7 Not reported |
| CAUSE OF ESKD (End Stage Kidney Disease)    (SRTR DATA CODES: HTN: 3034, 3040 DM: 3011, 3012, 3038, 3039, 3069, 3070, 3071  GN: 3000, 3001, 3002, 3003, 3004, 3005, 3006, 3041, 3043, 3067, 3068  PKD: 3008) | Patient's age in years at time of transplant evaluation. | Categorical | 1 Diabetes (T1DM or T2 DM)  2 Essential Hypertension  3 Renovascular Hypertension  3 Glomerulonephritis (Primary or secondary GN)  4 Interstitial nephritis/ Pyelonephritis  5 ADPKD (autosomal dominant kidney disease)  6 ARPKD (autosomal recessive kidney disease)  7 CAKUT/Cystic/Congenital/ Other AKUT (congenital anomalies of the kidney and urinary tract)  8 Neoplasms  9 Unknown  10 Other |
| HISTORY OF UTIs | Whether the patient has a history of UTIs. | Categorical | 1 Yes  2 No |
| HISTORY OF KIDNEY STONES | Whether the patient has a history of kidney stones. | Categorical | 1 Yes  2 No |
| HISTORY OF DRUG TREATED HYPOTENSION | Whether the patient has a history of drug treated hypotension. | Categorical | 1 Yes  2 No |
| HISTORY OF GOUT | Whether the patient has a history of gout. | Categorical | 1 Yes  2 No |
| HISTORY OF PRIOR TRANSPLANT | Whether the patient has a history of prior transplant. | Categorical | 1 Yes  2 No |
| What type of transplant | Descriptive information for what type of transplant. | Categorical | 1, Kidney 2, Liver 3, Kidney/Liver 4, Kidney/Pancreas xx, other |
| FAMILY HISTORY OF CKD (Chronic Kidney Disease) | Whether the patient has a history of ckd (chronic kidney disease). | Categorical | 1 Yes  2 No |
| HISTORY OF KIDNEY BIOPSY | Whether the patient has a history of kidney biopsy. | Categorical | 1 Yes  2 No |
| DIALYSIS ACCESS | Descriptive information for dialysis access. | Categorical | 1 PD catheter  2 AVF (Arterial Venous fistula)  3 AVG (Arterial Venous graft)  4 Permcath  5 Other, specify |
| RESIDUAL RENAL FUNCTION (are they making urine) | Descriptive information for residual renal function (are they making urine). | Categorical | 1 Yes  2 No |
| URINE OUTPUT (ML) | Descriptive information for urine output (ml). | Numerical |  |
| SYSTOLIC BLOOD PRESSURE | Descriptive information for systolic blood pressure. | Numerical |  |
| DIASTOLIC BLOOD PRESSURE | Descriptive information for diastolic blood pressure. | Numerical |  |
| PRETRANSPLANT EVALUATION DATE (first time by nephrology) | Descriptive information for pretransplant evaluation date (first time by nephrology). | Date |  |
| OSA (Obstructive Sleep Apnea) | Descriptive information for osa (obstructive sleep apnea). | Categorical | 1 Yes  2 No |
| TOTAL CHOLESTEROL | Descriptive information for total cholesterol. For each of the lab values we would like a pretransplant, posttransplant, and most recent value entered. Chose the two dates closest to the transplant date. | Numerical |  |
| LDL-C (mg/dL) | Descriptive information for ldl-c (mg/dl). | Numerical |  |
| HDL-C (mg/dL) | Descriptive information for hdl-c (mg/dl). | Numerical |  |
| TRIGLYCERIDES (mg/dL) | Descriptive information for triglycerides (mg/dl). | Numerical |  |
| CALCIUM (mg/dL) | Descriptive information for calcium (mg/dl). | Numerical |  |
| PHOSPHORUS (mg/dL) | Descriptive information for phosphorus (mg/dl). | Numerical |  |
| PTH (pg/mL) | Descriptive information for pth (pg/ml). | Numerical |  |
| VITAMIN D (pg/mL) - 25-hydroxyvitamin D | Descriptive information for vitamin d (pg/ml) - 25-hydroxyvitamin d. | Numerical |  |
| HEMOGLOBIN (g/dL) | Descriptive information for hemoglobin (g/dl). | Numerical |  |
| CYSTATIN | Descriptive information for cystatin. Will not be consistently ordered - if it is not listed the patient does not have it | Numerical |  |
| eGFR | Descriptive information for egfr. sometimes GFR will be listed as >60 or >90 both of these just mean normal so when it is listed >60 record 60 and when it is listed >90 record 90 | Numerical |  |
| MOST RECENT URINE PRO/CRE RATIO (g/g) | Descriptive information for most recent urine pro/cre ratio (g/g). | Numerical |  |
| If ratio is not listed: Most Recent Urine Protein | If you are collecting protein and creatinine seperately you need to make sure they were collected on the same day. This is most recent and only fill out if Pro/Cre ratio is not available for the most recent post transplant labs | Numerical |  |
| If ratio is not listed: Most Recent Urine Creatinine | Descriptive information for if ratio is not listed: most recent urine creatinine. This is most recent and only fill out if Pro/Cre ratio is not available for the most recent post transplant labs | Numerical |  |
| BMI | using height and weight at pre-transplant evaluation | Numerical |  |
| BSA | using height and weight at pre-transplant evaluation | Numerical |  |
| FINANCIAL CONCERNS | Descriptive information for financial concerns. | Categorical | 1 Yes  2 No |

Baseline Medications and Laboratory Values

| Field Label | Meaning | Data Type | Choices, Calculations, OR Slider Labels |
| --- | --- | --- | --- |
| Record ID | Autogenerated by REDCap. Will need to go back and "rename" to match the Patient ID | Numerical |  |
| Patient ID | This is the number assigned to each patient in the REDCap Masterlist | Numerical |  |
| ASA | Descriptive information for asa. | Categorical | 1 Yes 2 No |
| Antiplatlet | antiplatelet medications that the patient is **was on pre-transplant** | Categorical | 1, Clopidogrel 2, Ticagrelor 3, Ticlopidine 4, Prasugrel 5, Canagrelor 6, None xx, other |
| Anticoagulation Medication | anticoagulation medications that the patient is **was on pre-transplant** on | Categorical | 1, Enoxaparin 2, Apixaban 3, Dabigatran 4, Rivaroxaban 5, Warfarin 6, None xx, other |
| Beta Blocker Medications | betablockers that the patient is **was on pre-transplant** on | Categorical | 1, Metoprolol 2, Carvedilol 3, Bisoprolol 4, Sotalol 5, Atenolol 6, Esmolol 7, None xx, other |
| ACEi or ARB | ACE inhibitor or ARB that the patient is **was on pre-transplant** on | Categorical | 1, Lisinopril 2, Ramipril 3, Enalapril 4, Valsartan 5, Irbesartan 6, Telmisartan 7, Losartan 8, Entresto (Sacubitril-Valsartan) 9, None |
| CCB | Calcium channel blocker that the patient is **was on pre-transplant** on | Categorical | 1, Amlodipine 2, Nifedipine 3, Felodipine 4, Verapamil 5, Diltiazem 6, None xx, other |
| Hydralazine | was the patient on hydralazine **pre-transplant** | Categorical | 1 Yes 2 No |
| Nitrates | was the patient on nitrates **pre-transplant** | Categorical | 1 Yes 2 No |
| Diuretic | Diuretic that the patient was **previously** on pre-transplant | Categorical | 1, Furosemide 2, Hydrochlorothiazide 3, Spironolactone 4, Eplerenone 5, Bumetanide 6, Metolazone 7, Torsemide 8, Chlorothiazide 9, Chlorthalidone 10, None xx, other |
| BNP | some patients will not have this do we want it left blank - **closest date PRIOR to transplant** | Numerical |  |
| HBA1C | HBA1C lab value - **closest date PRIOR to transplant** | Numerical |  |

Current Medications and Laboratory Values

| Field Label | Meaning | Data Type | Choices, Calculations, OR Slider Labels |
| --- | --- | --- | --- |
| Record ID | Autogenerated by REDCap. Will need to go back and "rename" to match the Patient ID | Numerical |  |
| Patient ID | This is the number assigned to each patient in the REDCap Masterlist | Numerical |  |
| ASA | Descriptive information for asa. | Categorical | 1 Yes 2 No |
| Antiplatlet | antiplatelet medications that the patient is **currently** on | Categorical | 1, Clopidogrel 2, Ticagrelor 3, Ticlopidine 4, Prasugrel 5, Canagrelor 6, None xx, other |
| Anticoagulation Medication | anticoagulation medications that the patient is **currently** on | Categorical | 1, Enoxaparin 2, Apixaban 3, Dabigatran 4, Rivaroxaban 5, Warfarin 6, None xx, other |
| Beta Blocker Medications | betablockers that the patient is **currently** on | Categorical | 1, Metoprolol 2, Carvedilol 3, Bisoprolol 4, Sotalol 5, Atenolol 6, Esmolol 7, None xx, other |
| ACEi or ARB | ACE inhibitor or ARB that the patient is **currently** on | Categorical | 1, Lisinopril 2, Ramipril 3, Enalapril 4, Valsartan 5, Irbesartan 6, Telmisartan 7, Losartan 8, Entresto (Sacubitril-Valsartan) 9, None |
| CCB | Calcium channel blocker that the patient is **currently** on | Categorical | 1, Amlodipine 2, Nifedipine 3, Felodipine 4, Verapamil 5, Diltiazem 6, None xx, other |
| Hydralazine | is the patient **currently** on hydralazine | Categorical | 1 Yes 2 No |
| Nitrates | is the patient **currently** on nitrates | Categorical | 1 Yes 2 No |
| Diuretic | Diuretic that the patient is **currently** on | Categorical | 1, Furosemide 2, Hydrochlorothiazide 3, Spironolactone 4, Eplerenone 5, Bumetanide 6, Metolazone 7, Torsemide 8, Chlorothiazide 9, Chlorthalidone 10, None xx, other |
| BNP | some patients will not have this do we want it left blank - **Current** | Numerical |  |
| HBA1C | HBA1C lab value - **Current** | Numerical |  |

Left Heart Catheterization Pre-Transplant

| Field Label | Meaning | Data Type | Choices, Calculations, OR Slider Labels |
| --- | --- | --- | --- |
| Record IDs |  | Numerical |  |
| Date of Exam | Chart Review > Cardiac > CCL Cath etc./LHC or Left heart cath or balloon angioplasty | Date |  |
| Are there notable LHC findings | All further information on this form will be found in the same document . Not all patients will have a LHC | Categorical | 1, Yes 2, No |
| Which of the following vessel(s) are affected (select all that apply): |  | Categorical | 1, LAD \| 2, RCA \| 3, LCx \| 4, Diagonal 1 \| 5, Diagonal 2 \| 6, Obtuse marginal 1 \| 7, Obtuse marginal 2 \| 8, PDA |
| LAD | Location of stenosis | Categorical | 1, Proximal \| 2, Mid \| 3, Distal |
| Proximal LAD Degree of Stenosis % | Range of % stenosis | Categorical | 1, 0 to less than 29% \| 2, 30%-69% \| 3, 70% or greater |
| Proximal LAD Degree of Stenosis % | Exact % stenosis listed | Numerical |  |
| Mid LAD Degree of Stenosis % | Range of % stenosis | Categorical | 1, 0 to less than 29% \| 2, 30%-69% \| 3, 70% or greater |
| Mid LAD Degree of Stenosis % | Exact % stenosis listed | Numerical |  |
| Distal LAD Degree of Stenosis % | Range of % stenosis | Categorical | 1, 0 to less than 29% \| 2, 30%-69% \| 3, 70% or greater |
| Distal LAD Degree of Stenosis % | Exact % stenosis listed | Numerical |  |
| RCA | Location of stenosis | Categorical | 1, Proximal \| 2, Mid \| 3, Distal |
| Proximal RCA Degree of Stenosis % | Range of % stenosis | Categorical | 1, 0 to less than 29% \| 2, 30%-69% \| 3, 70% or greater |
| Proximal RCA Degree of Stenosis % | Exact % stenosis listed | Numerical |  |
| Mid RCA degree of Stenosis % | Range of % stenosis | Categorical | 1, 0 to less than 29% \| 2, 30%-69% \| 3, 70% or greater |
| Mid RCA Degree of Stenosis % | Exact % stenosis listed | Numerical |  |
| Distal RCA Degree of Stenosis % | Range of % stenosis | Categorical | 1, 0 to less than 29% \| 2, 30%-69% \| 3, 70% or greater |
| Distal RCA Degree of Stenosis % | Exact % stenosis listed | Numerical |  |
| LCx | Location of stenosis | Categorical | 1, Proximal \| 2, Mid \| 3, Distal |
| Proximal LCx Degree of Stenosis % | Range of % stenosis | Categorical | 1, 0 to less than 29% \| 2, 30%-69% \| 3, 70% or greater |
| Proximal LCx Degree of Stenosis % | Exact % stenosis listed | Numerical |  |
| Mid LCx Degree of Stenosis % | Range of % stenosis | Categorical | 1, 0 to less than 29% \| 2, 30%-69% \| 3, 70% or greater |
| Mid LCx Degree of Stenosis % | Exact % stenosis listed | Numerical |  |
| Distal LCx Degree of Stenosis % | Range of % stenosis | Categorical | 1, 0 to less than 29% \| 2, 30%-69% \| 3, 70% or greater |
| Distal LCx Degree of Stenosis % | Exact % stenosis listed | Numerical |  |
| Diagonal 1 | Location of stenosis | Categorical | 1, Proximal \| 2, Mid \| 3, Distal |
| Proximal Diagonal 1 Degree of Stenosis % | Range of % stenosis | Categorical | 1, 0 to less than 29% \| 2, 30%-69% \| 3, 70% or greater |
| Proximal Diagonal 1 Degree of Stenosis % | Exact % stenosis listed | Numerical |  |
| Mid Diagonal 1 Degree of Stenosis % | Range of % stenosis | Categorical | 1, 0 to less than 29% \| 2, 30%-69% \| 3, 70% or greater |
| Mid Diagonal 1 Degree of Stenosis % | Exact % stenosis listed | Numerical |  |
| Distal Diagonal 1 Degree of Stenosis % | Range of % stenosis | Categorical | 1, 0 to less than 29% \| 2, 30%-69% \| 3, 70% or greater |
| Distal Diagonal 1 Degree of Stenosis % | Exact % stenosis listed | Numerical |  |
| Diagonal 2 | Location of stenosis | Categorical | 1, Proximal \| 2, Mid \| 3, Distal |
| Proximal Diagonal 2 Degree of Stenosis % | Range of % stenosis | Categorical | 1, 0 to less than 29% \| 2, 30%-69% \| 3, 70% or greater |
| Proximal Diagonal 2 Degree of Stenosis % | Exact % stenosis listed | Numerical |  |
| Mid Diagonal 2 Degree of Stenosis % | Range of % stenosis | Categorical | 1, 0 to less than 29% \| 2, 30%-69% \| 3, 70% or greater |
| Mid Diagonal 2 Degree of Stenosis % | Exact % stenosis listed | Numerical |  |
| Distal Diagonal 2 Degree of Stenosis % | Range of % stenosis | Categorical | 1, 0 to less than 29% \| 2, 30%-69% \| 3, 70% or greater |
| Distal Diagonal 2 Degree of Stenosis % | Exact % stenosis listed | Numerical |  |
| Obtuse Marginal 1 | Location of stenosis | Categorical | 1, Proximal \| 2, Mid \| 3, Distal |
| Proximal Obtuse Marginal 1 Degree of Stenosis % | Range of % stenosis | Categorical | 1, 0 to less than 29% \| 2, 30%-69% \| 3, 70% or greater |
| Proximal Obtuse Marginal 1 Degree of Stenosis % | Exact % stenosis listed | Numerical |  |
| Mid Obtuse Marginal 1 Degree of Stenosis % | Range of % stenosis | Categorical | 1, 0 to less than 29% \| 2, 30%-69% \| 3, 70% or greater |
| Mid Obtuse Marginal 1 Degree of Stenosis % | Exact % stenosis listed | Numerical |  |
| Distal Obtuse Marginal 1 Degree of Stenosis % | Range of % stenosis | Categorical | 1, 0 to less than 29% \| 2, 30%-69% \| 3, 70% or greater |
| Distal Obtuse Marginal 1 Degree of Stenosis % | Exact % stenosis listed | Numerical |  |
| Obtuse Marginal 2 | Location of stenosis | Categorical | 1, Proximal \| 2, Mid \| 3, Distal |
| Proximal Obtuse Marginal 2 Degree of Stenosis % | Range of % stenosis | Categorical | 1, 0 to less than 29% \| 2, 30%-69% \| 3, 70% or greater |
| Proximal Obtuse Marginal 2 Degree of Stenosis % | Exact % stenosis listed | Numerical |  |
| Mid Obtuse Marginal 2 Degree of Stenosis % | Range of % stenosis | Categorical | 1, 0 to less than 29% \| 2, 30%-69% \| 3, 70% or greater |
| Mid Obtuse Marginal 2 Degree of Stenosis % | Exact % stenosis listed | Numerical |  |
| Distal Obtuse Marginal 2 Degree of Stenosis % | Range of % stenosis | Categorical | 1, 0 to less than 29% \| 2, 30%-69% \| 3, 70% or greater |
| Distal Obtuse Marginal 2 Degree of Stenosis % | Exact % stenosis listed | Numerical |  |
| Posterior Descending Artery | Location of stenosis | Categorical | 1, Proximal \| 2, Mid \| 3, Distal |
| Proximal PDA Degree of Stenosis % | Range of % stenosis | Categorical | 1, 0 to less than 29% \| 2, 30%-69% \| 3, 70% or greater |
| Proximal PDA Degree of Stenosis % | Exact % stenosis listed | Numerical |  |
| Mid PDA Degree of Stenosis % | Range of % stenosis | Categorical | 1, 0 to less than 29% \| 2, 30%-69% \| 3, 70% or greater |
| Mid PDA Degree of Stenosis % | Exact % stenosis listed | Numerical |  |
| Distal PDA Marginal 2 Degree of Stenosis % | Range of % stenosis | Categorical | 1, 0 to less than 29% \| 2, 30%-69% \| 3, 70% or greater |
| Distal PDA Marginal 2 Degree of Stenosis % | Exact % stenosis listed | Numerical |  |

Baseline Echo

| Field Label | Meaning | Located | Data Type |
| --- | --- | --- | --- |
| Record ID | Descriptive information for record id. |  | Numerical |
| Patient ID | Descriptive information for patient id. | Input record ID number that is associated with the MRN assigned | Numerical |
| Echo ID | Descriptive information for echo id. |  |  |
| Echo Timepoint | Descriptive information for echo timepoint. | Listed under "exam date" and should be ex: 15:33 | Numerical |
| Date of Echo | Descriptive information for date of echo. | Listed under "exam date" and should be ex: DD/MM/YYYY | Numerical |
| LVEF | left ventricle ejection fraction | Listed under "findings" and should be a %, ex: LVEF 55% | Numerical |
| RV Function | Right ventricular function | Listed under "findings" under the "right ventricle" section | Categorical |
| TAPSE (cm) | Tricuspid Annular plane systolic excursion | Listed under "measurements" under "m mode measurement" section | Numerical |
| IVSd (cm) | Interventricular septum diameter | Listed under "measurements" under "IVS Diastolic Thickness MM" | Numerical |
| LVPWd | end diastolic left ventricular posterior wall thickness | Listed under "measurements" under "LVPW Diastolic Thickness MM" | Numerical |
| MR severity | Mitral regurgitation severity | Listed under "findings" under the "mitral valve" section | Categorical |
| TR Severity | Tricuspid regurgitation severity | Listed under "findings" under the "tricuspid valve" section | Categorical |
| RSVP | Right ventricular systolic pressure or PASP (pulmonary artery systolic pressure) | Listed under "findings" under the "tricuspid valve" section or under the "doppler measurements" | Numerical |
| TR Peak Gradient | Tricuspid regurgitation peak gradient | Listed under "measurements" under the tricuspid valve section | Numerical |
| AS severity | Aortic stenosis severity | Listed under "findings" under the "aortic valve" section | Categorical |
| AR severity | Aortic regurgitation severity | Listed under "findings" under the "aortic valve" section | Categorical |
| LVEDVI | Left ventricular end diastolic volume indexed. Left ventricular end diastolic volume / body surface area |  | Numerical |
| LV Mass | Left ventricular mass | Listed under "measurements" under "LV Mass Index MM" | Numerical |
| LVESVI | Left ventricular end diastolic volume indexed. Left ventricular end systolic volume / body surface area |  | Numerical |
| Pericardial Effusion | Descriptive information for pericardial effusion. | Listed under "findings" under the "pericardium" section | Categorical |
| RA area | Right atrium area | Listed under "measurements" under apical 2D dimensions | Numerical |
| LA Volume | left atrium volume | Listed under "measurements" under "LA Volume" | Numerical |
| LA max 4ch area | left atrium max area in 4 chamber view |  | Numerical |
| LA max 2ch area | left atrium max area in 2 chamber view |  | Numerical |
| LA max 2ch length | left atrium max length in 2 chamber view |  | Numerical |
| LA max 4ch length | left atrium max length in 4 chamber view |  | Numerical |
| LAV max | maximum left atrial volume |  | Numerical |
| LAV max index | maximum left atrial volume index to body surface area |  | Numerical |
| LA min 4ch area | minimum left atrial volume area in 4 chamber view |  | Numerical |
| LA min 4ch length | minimum left atrial volume length in 4 chamber view |  | Numerical |
| LA min 2ch area | minimum left atrial volume area in 2 chamber view |  | Numerical |
| LA min 2ch length | minimum left atrial volume length in 2 chamber view |  | Numerical |
| LAV min | minimum left atrial volume |  | Numerical |
| LAV min index | minimum left atrial volume index to body surface area |  | Numerical |
| LA preA 4ch area | pre-atrial contraction left atrium volume 4 chamber view |  | Numerical |
| LA preA 4ch length | pre-atrial contraction left atrium length 4 chamber view |  | Numerical |
| LA preA 2ch area | pre-atrial contraction left atrium volume 2 chamber view |  | Numerical |
| LA preA 2ch length | pre-atrial contraction left atrium length 2 chamber view |  | Numerical |
| LAV preA | left atrial volume before atrial systole |  | Numerical |
| LAV preA index | Descriptive information for lav preA index. |  | Numerical |
| LA EF Passive (max - preA)/max | Descriptive information for la ef passive (max - prea)/max. |  | Numerical |
| Active (preA-min)/preA | Descriptive information for active (prea-min)/prea. |  | Numerical |
| LA EF Total (max-min)/max | Descriptive information for la ef total (max-min)/max. |  | Numerical |
| LA expansion index % (max - min)/min) | Descriptive information for la expansion index % (max - min)/min). |  | Numerical |
| LA reservoir strain 2ch | left atrial reservoir in 2 chamber view. Difference between conduit and contraction measurement | Measured using tomtec through synapse | Numerical |
| LA reservoir strain 4ch | left atrial reservoir in 4 chamber view. Difference between conduit and contraction measurement | Measured using tomtec through synapse | Numerical |
| LA contractile strain 2ch | left atrial contraction function 2 chamber view. maximum left atrial contraction | Measured using tomtec through synapse | Numerical |
| LA contractile strain 4ch | left atrial contraction function 4 chamber view. maximum left atrial contraction | Measured using tomtec through synapse | Numerical |
| LA conduit strain 2ch | left atrial conduit function 2 chamber view. first positive peak | Measured using tomtec through synapse | Numerical |
| LA conduit strain 4ch | left atrial conduit function 2 chamber view. first positive peak | Measured using tomtec through synapse | Numerical |
| RV FW Basal | Right ventricle free wall basal segment |  | Numerical |
| RV FW Mid | Right ventricle free wall mid segment |  | Numerical |
| RV FW Apical | Right ventricle free wall apical segment |  | Numerical |
| RV FW Average Strain | Right ventricle free wall average strain |  | Numerical |

Echo Stress Test

| Field Label | Meaning | Located | Data Type | Choices, Calculations, OR Slider Labels |
| --- | --- | --- | --- | --- |
| Record ID | Descriptive information for record id. |  |  |  |
| Patient ID | Descriptive information for patient id. | Input record ID number that is associated with the MRN assigned | Numerical |  |
| Type | Descriptive information for type. |  | Categorical | 1, Pharmacological \| 2, Exercise |
| Stress Echo Time Point | Descriptive information for stress echo time point. | Listed under "exam date" and should be ex: 15:33 | Numerical | 1, initial transplant evaluation \| 2, surveillance on the waiting list \| 3, clinical evaluation on list (patient became unstable) \| 4, clinical evaluation after transplant |
| Stress Echo Date | Descriptive information for stress echo date. | Listed under "exam date" and should be ex: DD/MM/YYYY | Numeric |  |
| Electrocardiogram (ECG) Changes | Descriptive information for electrocardiogram (ecg) changes. | Listed under "ECG analysis" and under "Stress ECG" | Categorical | 1, ST Depression \| 2, ST Elevation \| 3, None |
| Exercise Duration | Descriptive information for exercise duration. | Listed under "stress results" and under "Exercise Duration (min:sec)" | Numerical |  |
| Wall motion abnormalities | Descriptive information for wall motion abnormalities. | Listed under "echo findings" | Categorical |  |
| Number of segments with Wall Motion Abnormalities | Descriptive information for number of segments with wall motion abnormalities. | Listed under "echo findings" | Numerical |  |
| Target HR achieved | Descriptive information for target hr achieved. | Listed under "stress results" and under "stress summary" | Categorical |  |
| METs | Descriptive information for mets. | Listed under "stress results" and under "METS" | Numerical |  |
| Exercise Capacity | Descriptive information for exercise capacity. ID protocol used - Bruce or modified Bruce for bruce - https://www.omnicalculator.com/sports/bruce-protocol-mets enter age, sex, time Results will come out under metabolic results Look at MET mex Use table below metabolic results to categorize |  | Categorical | 1, Poor \| 2, Fair \| 3, Average \| 4, Above average \| 5, Excellent |
| BP response to exercise | Descriptive information for bp response to exercise. | Listed under "stress results" and under "BP response" | Categorical | 1, normal \| 2, hypotension \| 3, hypertensive |

Nuclear Stress Test

| Field Label | Meaning | Located | Data Type | Choices, Calculations, OR Slider Labels |
| --- | --- | --- | --- | --- |
| Record ID | Descriptive information for record id. |  |  |  |
| Patient ID | Descriptive information for patient id. | Input record ID number that is associated with the MRN assigned | Numerical |  |
| Test Time Point | Descriptive information for test time point. |  |  | 1, initial transplant evaluation \| 2, surveillance on the waiting list \| 3, clinical evaluation on list (patient became unstable) \| 4, clinical evaluation after transplant |
| Date of Nuclear Stress Test | Descriptive information for date of nuclear stress test. | Listed under "exam date" or "study date" MM/DD/YYYY | Numerical |  |
| Types | Descriptive information for types. |  | Categorical | 1, Exercise \| 2, Pharmacologic |
| Number of Hypoperfused Segments at Rest | Descriptive information for number of hypoperfused segments at rest. | Listed under "stress information" and "baseline ecg" and is described by decreased activity in a coronary vessel | Numerical |  |
| Number of Hypoperfused Segments with Stress | Descriptive information for number of hypoperfused segments with stress. | Listed under "findings" and is described by decreased activity in a coronary vessel | Numerical |  |
| Ejection Fraction | Descriptive information for ejection fraction. | Listed under "findings" and is reported as a percent ex: 55% | Numerical |  |
| Total Calcium Score | Descriptive information for total calcium score. | Listed under "Calcium score" | Numerical |  |
| Lm Calcium Score | Descriptive information for left main calcium score. | Listed under "Calcium score" | Numerical |  |
| LAD Calcium Score | Descriptive information for left anterior descending calcium score. | Listed under "Calcium score" | Numerical |  |
| LCX Calcium Score | Descriptive information for left circumflex calcium score. | Listed under "Calcium score" | Numerical |  |
| RCA Calcium Score | Descriptive information for right coronary artery calcium score. | Listed under "Calcium score" | Numerical |  |

Right Heart Catheterization

| Field Label | Meaning | Data Type | Choices, Calculations, OR Slider Labels |
| --- | --- | --- | --- |
| Record ID |  |  |  |
| Date of Right Heart Cath | Listed under "procedure date" MM/DD/YYYY | Numerical |  |
| Mean Right Atrial Pressure (RAP) | Listed under "pressure phases: baseline" and "RA pressures" and under "mean" | Numerical |  |
| Systolic Pulmonary Artery Pressure (PAP) | Listed under "pressure phases: baseline" and "PA pressures" and under "systolic" | Numerical |  |
| Diastolic Pulmonary Artery Pressure (PAP) | Listed under "pressure phases: baseline" and "PA pressures" and under "diastolic" | Numerical |  |
| Mean Pulmonary Artery Pressure (PAP) | Listed under "pressure phases: baseline" and "PA pressures" and under "mean" | Numerical |  |
| Mean Pulmonary Capillary Wedge Pressure (PCWP) | Listed under "pressure phases: baseline" and "PCW pressures" and under "mean" | Numerical |  |
| Cardiac Output | Listed under "valve measurements" and under "Fick C.O" or under "cardiac output phases: baseline" and "Fick C.O" | Numerical |  |
| Cardiac Index | Listed under "cardiac output phases: baseline" and "Fick C.I" | Numerical |  |
| Mixed Venous Oxygen Saturation | MVO2. If you do not see MVO2 then report PA oxygen saturation | Numerical |  |
| Is the above value PA or MVO2 |  | Categorical | 1, PA 2, MVO2 |
| Systemic Vascular Resistance |  | Numerical |  |
| Pulmonary Vascular Resistance | Listed under "right heart cath findings" and "PVR" or "resistance results phases: baseline" and "PVR" | Numerical |  |
| PAPi | Listed under "right heart cath findings" and "PAPi" | Numerical |  |

Outcomes

| Field Label | Meaning | Choices, Calculations, OR Slider Labels |
| --- | --- | --- |
| Record ID |  |  |
| Cardiovascular Death | Definition of Cardiovascular Death: Death from MI, arrhythmias, stroke, vascular causes (peripheral artery disease, ruptured aneurysm, aortic dissection), and complications from cardiovascular procedures (CABG, PCI or balloon angioplasty, dissection repair, etc) | When you open up MRN it should show "deceased" and grayed out image in top left corner near name. Then read the last discharge summary. |
| Date of Cardiovascular Death | Descriptive information for date of cardiovascular death. |  |
| Non-Fatal Myocardial Infarction | Definittion of MI: STEMI, NSTEMI. "Chest pain", "NSTEMI", "STEMI" |  |
| Was the Non-Fatal MI STEMI or NSTEMI | Non-fatal myocardial infarction classified as a ST elevation myocardial infarction or a non-ST elevation myocardial infarction |  |
| Date of Non-Fatal Myocardial Infarction | Descriptive information for date of non-fatal myocardial infarction. |  |
| Ischemic Stroke | Descriptive information for ischemic stroke. "Stroke" |  |
| Date of Ischemic Stroke | Descriptive information for date of ischemic stroke. |  |
| Hemorrhagic Stroke | Descriptive information for hemorrhagic stroke. "Stroke" |  |
| Date of Hemorrhagic Stroke | Descriptive information for date of hemorrhagic stroke. |  |
| Hospitalization for Unstable Angina | Descriptive information for hospitalization for unstable angina. "Angina", "Chest pain", "Unstable Angina" | It is possible for chest pain to be listed as a problem in a note and it NOT be cardiac in nature. A way to confirm if you are unsure: Was their troponin levels elevated? Was Cardiology consulted? Mention of EKG changes? Did the note state it was reproducible chest pain ? (less likely cardiac). If you are unsure, flag this patient in the issues tab |
| Date of Hospitalization for Unstable Angina | Descriptive information for date of hospitalization for unstable angina. |  |
| Coronary Revascularization Procedures (Percutaneous Coronary Intervention) | PCI, "drug eluting stent", "stent" |  |
| Date of Coronary Revascularization Procedures (Percutaneous Coronary Intervention) | Descriptive information for date of percutaneous intervention | Patient's would have a left heart cath typically or STEMI or NSTEMI that leads to an acute intervention; within the cath report or procedure report, you will find if there was PCI done |
| Coronary Revascularization Procedures (Coronary Artery Bypass Grafting) | Patient's would have a left heart cath typically or STEMI or NSTEMI that leads to an acute intervention; Cardiothoracic surgery would have been consulted and there would be a procedure report. "CABG", "multivessel disease", "Coronary Artery Bypass Grafting" |  |
| Date of Coronary Revascularization Procedures (Coronary Artery Bypass Grafting) | Descriptive information for date of CABG |  |
| Hospitalization for Heart Failure | Descriptive information for hospitalization of heart failure. "HFrEF", "HFpEF", "Heart failure exacerbation", "volume overload", "dyspnea", "dyspnea on exertion", "DOE", "Congestive heart failure exacerbation", "diastolic heart failure exacerbation", "systolic heart failure exacerbation" | Patients would have been admitted because of an ACUTE exacerbation of heart failure |
| Date of Hospitalization for Heart Failure | Descriptive information for date of heart failure exacerbation |  |
| Major Arrhythmic Events (e.g., Ventricular Tachycardia, Ventricular Fibrillation) | Only include hospitalizations where the patient was either admitted for OR their hospital stay was complicated by SUSTAINED (>/= 30 seconds) ventricular tachycardia or ventricular fibrillation. "Ventricular fibrillation", "Ventricular Tachycardia", "VT", "SVT", "Sustained VT" | Cut off for sustained is greater than or equal to 30 seconds; Remember that we are not interested in non-sustained ventricular tachycardia's. If the run is reported in "beats" and not in "seconds" use the following judgment - if greater than or equal to 50 beats this is considered "sustained", please FLAG ALL PATIENTS THAT REPORT BEATS INSTEAD OF SECONDS; we will confirm this after your entry |
| Date of Major Arrhythmic Events (e.g., Ventricular Tachycardia, Ventricular Fibrillation) | Descriptive information for date of major arrhythmic event |  |
| All-Cause Mortality (including cardiovascular and non-cardiovascular causes) | Death by any cause | When you open up MRN it should show "deceased" and grayed out image in top left corner near name. Then read the last discharge summary. |
| Date of All-Cause Mortality (including cardiovascular and non-cardiovascular causes) | Descriptive information for death by any cause |  |

**Supplemental Appendix 3:**

from autogluon.tabular import TabularDataset, TabularPredictor

from sklearn.model_selection import train_test_split

from sklearn.preprocessing import StandardScaler

from missforest import MissForest

import matplotlib.pyplot as plt

import pandas as pd

import numpy as np

import shap

from sklearn.metrics import roc_curve, auc, roc_auc_score, f1_score, roc_auc_score, precision_score, recall_score, precision_recall_curve, average_precision_score

from sklearn.preprocessing import StandardScaler

from sklearn.model_selection import StratifiedKFold

#70% training and 30% testing dataset generation

def sksplit():

    x = []

    y = []

    df = pd.read_csv('initial_data_encoded.csv')

    x = df.iloc[:, :-1]

    y = df.iloc[:, -1]

    x_train, x_test, y_train, y_test = train_test_split(x, y, test_size=0.3, random_state=42)

    train = pd.concat([x_train, y_train], axis=1)

    test = pd.concat([x_test, y_test], axis=1)

    train.to_csv('train.csv', index=False)

    test.to_csv('test.csv', index=False)

#MissForest data imputation

def imp():

    train = pd.read_csv('train.csv')

    test = pd.read_csv('test.csv')

    train_without_mace = train.drop(columns=['MACE'])

    test_without_mace = test.drop(columns=['MACE'])

    categorical_columns = ['Sex Male', 'Race Black', 'Race White', 'Hemodialysis', 'Peritoneal Dialysis', 'Obstructive Sleep Apnea', 'Type 2 Diabetes Mellitus', 'Coronary Artery Disease', 'Tobacco Use', 'Myocardial Infarction', 'Cerebrovascular Accident', 'Unstable Angina', 'Heart Failure', 'Arrythmia', 'Mitral Regurgitation', 'Tricuspid Regurgitation', 'Aortic Regurgitation', 'Troponin I elevation', 'Transplant Number', 'Former Smoker', 'Current Smoker']

    mf = MissForest(categorical=categorical_columns)

    train_imp = mf.fit_transform(train_without_mace)

    test_imp = mf.transform(test_without_mace)

    train_imp = pd.DataFrame(train_imp, columns=train_without_mace.columns)

    test_imp = pd.DataFrame(test_imp, columns=test_without_mace.columns)

    train_imp['MACE'] = train['MACE'].values

    test_imp['MACE'] = test['MACE'].values

    train_imp.to_csv('train_imp.csv', index=False)

    test_imp.to_csv('test_imp.csv', index=False)

#Data scaling

train = pd.read_csv('train_imp.csv')

    test = pd.read_csv('test_imp.csv')

    train_without_mace = train.drop(columns=['MACE'])

    test_without_mace = test.drop(columns=['MACE'])

    categorical_columns = ['Sex Male', 'Race Black', 'Race White', 'Hemodialysis', 'Peritoneal Dialysis', 'Obstructive Sleep Apnea', 'Type 2 Diabetes Mellitus', 'Coronary Artery Disease', 'Tobacco Use', 'Myocardial Infarction', 'Cerebrovascular Accident', 'Unstable Angina', 'Heart Failure', 'Arrythmia', 'Mitral Regurgitation', 'Tricuspid Regurgitation', 'Aortic Regurgitation', 'Troponin I elevation', 'Transplant Number', 'Former Smoker', 'Current Smoker']

    train_cat = train_without_mace[categorical_columns]

    test_cat = test_without_mace[categorical_columns]

    train_numeric = train_without_mace.drop(columns=categorical_columns)

    test_numeric = test_without_mace.drop(columns=categorical_columns)

    scaler = StandardScaler()

    train_scaled = scaler.fit_transform(train_numeric)

    test_scaled = scaler.transform(test_numeric)

    train_scaled_df = pd.DataFrame(train_scaled, columns=train_numeric.columns)

    test_scaled_df = pd.DataFrame(test_scaled, columns=test_numeric.columns)

    train_scaled_df = pd.concat([train_scaled_df, train_cat], axis=1)

    test_scaled_df = pd.concat([test_scaled_df, test_cat], axis=1)

    train_scaled_df['MACE'] = train['MACE'].values

    test_scaled_df['MACE'] = test['MACE'].values

    train_scaled_df.to_csv('train_scaled.csv', index=False)

    test_scaled_df.to_csv('test_scaled.csv', index=False)

#Feature selection

from sklearn.inspection import permutation_importance

from lightgbm import LGBMClassifier

def fsel_permutation_lgbm_cv(n_splits=5, top_k=10):

    data = TabularDataset('train_imp.csv')

    label = "MACE"

    skf = StratifiedKFold(n_splits=n_splits, shuffle=True, random_state=42)

    fold_features = []

    X = data.drop(columns=[label])

    y = data[label]

    feature_names = X.columns.tolist()

    fold_num = 1

    for train_idx, val_idx in skf.split(X, y):

        print(f"Processing Fold {fold_num}...")

        X_train = X.iloc[train_idx]

        y_train = y.iloc[train_idx]

        X_val = X.iloc[val_idx]

        y_val = y.iloc[val_idx]

        scaler = StandardScaler()

        X_train_scaled = scaler.fit_transform(X_train)

        X_val_scaled = scaler.transform(X_val)

        model = LGBMClassifier(

            n_estimators=1000,

            learning_rate=0.01,

            random_state=42,

            n_jobs=-1,

            importance_type='gain'

        )

        model.fit(

            X_train_scaled, y_train,

            eval_set=[(X_val_scaled, y_val)],

            eval_metric='aucpr'

        )

        result = permutation_importance(

            model, X_val_scaled, y_val,

            n_repeats=10,

            scoring='average_precision',

            random_state=42,

            n_jobs=-1

        )

        importances = result.importances_mean

        top_indices = np.argsort(importances)[::-1][:top_k]

        selected = [feature_names[i] for i in top_indices if importances[i] > 0]

        fold_features.append({

            'Fold': fold_num,

            'Selected_Features': selected

        })

        fold_num += 1

    features_df = pd.DataFrame(fold_features)

    features_df.to_csv("nested_permutation_lightgbm_folds.csv", index=False)

#AutoGluon Tabular Predictor

def tabular():

    train_data = TabularDataset('train_fsel.csv')

    test_data = TabularDataset('test_fsel.csv')

    label = "MACE"

    predictor = TabularPredictor(label=label, problem_type='binary', eval_metric='average_precision').fit(train_data, auto_stack=True, presets='best_quality')

    predictor.fit_summary()

    importance_data = predictor.feature_importance(test_data, subsample_size=None, num_shuffle_sets=5, confidence_level=0.95)

    importance_data.index = [f"{feature} (p={round(p, 3)})"

    for feature, p in zip(importance_data.index, importance_data['p_value'])]

    importance_data.to_csv('importance_'+label+'.csv', encoding='UTF-8')

    leaderboard_data = predictor.leaderboard(test_data, extra_metrics=['average_precision','roc_auc', 'accuracy'], silent=True)

    leaderboard_data.to_csv('leaderboard_'+label+'.csv', encoding='UTF-8')

#Metric calculations

def bootstrap_auc_ci(y_true, y_proba, n_bootstraps=5000, alpha=0.05):

    rng = np.random.RandomState(42)

    bootstrapped_scores = []

    for _ in range(n_bootstraps):

        indices = rng.choice(range(len(y_proba)), size=len(y_proba), replace=True)

        if len(np.unique(y_true.iloc[indices])) < 2:

            continue

        score = roc_auc_score(y_true.iloc[indices], y_proba[indices])

        bootstrapped_scores.append(score)

    sorted_scores = np.sort(bootstrapped_scores)

    ci_low = sorted_scores[int((alpha / 2) * len(sorted_scores))]

    ci_high = sorted_scores[int((1 - alpha / 2) * len(sorted_scores))]

    auc_val = roc_auc_score(y_true, y_proba)

    return ci_low, auc_val, ci_high

def find_best_threshold(y_true, y_proba):

    thresholds = np.linspace(0, 1, 10001)

    best_f1 = 0

    best_threshold = 0.5

    for thresh in thresholds:

        y_pred = (y_proba >= thresh).astype(int)

        f1 = f1_score(y_true, y_pred, zero_division=0)

        if f1 > best_f1:

            best_f1 = f1

            best_threshold = thresh

    return best_threshold, best_f1

def find_best_threshold(y_true, y_proba):

    precisions, recalls, thresholds = precision_recall_curve(y_true, y_proba)

    f1_scores = 2 * (precisions * recalls) / (precisions + recalls + 1e-8)

    best_idx = np.argmax(f1_scores)

    return thresholds[best_idx], f1_scores[best_idx]

def bootstrap_metric_ci(y_true, y_proba, metric_func, n_bootstrap=1000, alpha=0.05):

    rng = np.random.default_rng(42)

    boot_metrics = []

    for _ in range(n_bootstrap):

        indices = rng.integers(0, len(y_true), len(y_true))

        y_true_boot = y_true.iloc[indices]

        y_proba_boot = y_proba[indices]

        if metric_func.__name__ in ["precision_score", "recall_score", "f1_score"]:

            threshold, _ = find_best_threshold(y_true_boot, y_proba_boot)

            y_pred_boot = (y_proba_boot >= threshold).astype(int)

            score = metric_func(y_true_boot, y_pred_boot, zero_division=0)

        elif metric_func.__name__ == "auc":

            precisions, recalls, _ = precision_recall_curve(y_true_boot, y_proba_boot)

            score = auc(recalls, precisions)

        else:

            score = metric_func(y_true_boot, y_proba_boot)

        boot_metrics.append(score)

    lower = np.percentile(boot_metrics, 100 * alpha / 2)

    upper = np.percentile(boot_metrics, 100 * (1 - alpha / 2))

    return lower, np.mean(boot_metrics), upper

def evaluation():

    predictor = TabularPredictor.load('AutogluonModels/ag-20250720_195507')

    test_data = pd.read_csv("test_fsel.csv")

    label = predictor.label

    test_y = test_data[label]

    test_x = test_data.drop(columns=[label])

    leaderboard = pd.read_csv("leaderboard_MACE.csv")

    models = leaderboard["model"].tolist()

    top_models = models[:10]

    results = []

    for model in top_models:

        y_proba = predictor.predict_proba(test_x, model=model)[1]

        threshold, best_f1 = find_best_threshold(test_y, y_proba)

        y_pred = (y_proba >= threshold).astype(int)

        roc_auc = roc_auc_score(test_y, y_proba)

        precision = precision_score(test_y, y_pred, zero_division=0)

        recall = recall_score(test_y, y_pred, zero_division=0)

        precisions, recalls, _ = precision_recall_curve(test_y, y_proba)

        pr_auc = auc(recalls, precisions)

        roc_ci_low, _, roc_ci_high = bootstrap_metric_ci(test_y, y_proba, roc_auc_score)

        f1_ci_low, _, f1_ci_high = bootstrap_metric_ci(test_y, y_proba, f1_score)

        precision_ci_low, _, precision_ci_high = bootstrap_metric_ci(test_y, y_proba, precision_score)

        recall_ci_low, _, recall_ci_high = bootstrap_metric_ci(test_y, y_proba, recall_score)

        pr_auc_ci_low, _, pr_auc_ci_high = bootstrap_metric_ci(test_y, y_proba, auc)

        results.append({

            "model": model,

            "roc_auc": roc_auc,

            "roc_auc_CI_low": roc_ci_low,

            "roc_auc_CI_high": roc_ci_high,

            "f1_score": best_f1,

            "f1_CI_low": f1_ci_low,

            "f1_CI_high": f1_ci_high,

            "precision": precision,

            "precision_CI_low": precision_ci_low,

            "precision_CI_high": precision_ci_high,

            "recall": recall,

            "recall_CI_low": recall_ci_low,

            "recall_CI_high": recall_ci_high,

            "pr_auc": pr_auc,

            "pr_auc_CI_low": pr_auc_ci_low,

            "pr_auc_CI_high": pr_auc_ci_high,

            "optimal_threshold": threshold

        })

    results_df = pd.DataFrame(results)

    results_df.to_csv("metrics.csv", index=False)

#SHAP and AUROC generation

def interpret():

    test = pd.read_csv('test_fsel.csv')

    y_test = test['MACE']

    test = test.drop(columns=['MACE'])

    predictor = TabularPredictor.load('AutogluonModels/ag-20250720_195507')

    model_name = 'CatBoost_r128_BAG_L1'

    sampled_test = test.sample(n=100, random_state=42)

    predict_fn = lambda x: predictor.predict_proba(x, model=model_name)[1]

    explainer = shap.Explainer(predict_fn, test)

    shap_values = explainer(sampled_test)

    shap.plots.beeswarm(shap_values, max_display=30)

    shap.plots.waterfall(shap_values[5], max_display=10)

    r_probs = pd.read_csv("r_preds.csv")

    h_probs = pd.read_csv("soveri.csv")

    y_score_h = h_probs['prob']

    y_score_r = r_probs['prob_r']

    y_true_r = r_probs['MACE']

    y_true_h = h_probs['MACE']

    y_pred_proba = predictor.predict_proba(test, model=model_name)[1]

    fpr_ag, tpr_ag, _ = roc_curve(y_test, y_pred_proba)

    roc_auc_ag = auc(fpr_ag, tpr_ag)

    ci_low_ag, auc_val_ag, ci_high_ag = bootstrap_auc_ci(y_test, y_pred_proba)

    fpr_r, tpr_r, _ = roc_curve(y_true_r, y_score_r)

    roc_auc_r = auc(fpr_r, tpr_r)

    ci_low_r, auc_val_r, ci_high_r = bootstrap_auc_ci(y_true_r, y_score_r)

    fpr_h, tpr_h, _ = roc_curve(y_true_h, y_score_h)

    roc_auc_h = auc(fpr_h, tpr_h)

    ci_low_h, auc_val_h, ci_high_h = bootstrap_auc_ci(y_true_h, y_score_h)

    plt.figure(figsize=(10, 8))

    plt.plot(fpr_ag, tpr_ag, color='blue', lw=2, label=f'CatBoost_r128_BAG_L1 (AUC = {roc_auc_ag:.3f} CI {ci_low_ag:.3f} - {ci_high_ag:.3f})')

    plt.plot(fpr_r, tpr_r, color='green', lw=2, label=f'Logistic Regression (AUC = {roc_auc_r:.3f} CI {ci_low_r:.3f} - {ci_high_r:.3f})')

    plt.plot(fpr_h, tpr_h, color='orange', lw=2, label=f'Historical Model (AUC = {roc_auc_h:.3f} CI {ci_low_h:.3f} - {ci_high_h:.3f})')

    plt.plot([0, 1], [0, 1], color='gray', linestyle='--')

    plt.xlim([0.0, 1.0])

    plt.ylim([0.0, 1.05])

    plt.xlabel('False Positive Rate')

    plt.ylabel('True Positive Rate')

    plt.title('ROC Curve Comparison')

    plt.legend(loc="lower right")

    plt.grid(True)

    plt.tight_layout()

    plt.show()

#AUPRC generation

def bootstrap_f1_threshold(y_true, y_probs, n_bootstraps=1000, seed=123):

    f1_boot, thresh_boot = [], []

    rng = np.random.default_rng(seed)

    for _ in range(n_bootstraps):

        idx = rng.choice(len(y_true), len(y_true), replace=True)

        yb_true = y_true[idx]

        yb_probs = y_probs[idx]

        p, r, t = precision_recall_curve(yb_true, yb_probs)

        f1 = 2 * (p * r) / (p + r + 1e-8)

        idx_max = np.argmax(f1)

        f1_boot.append(f1[idx_max])

        if idx_max < len(t):

            thresh_boot.append(t[idx_max])

    return np.percentile(f1_boot, [2.5, 97.5]), np.percentile(thresh_boot, [2.5, 97.5])

def safe_auc(x, y):

    sorted_indices = np.argsort(x)

    x_sorted = x[sorted_indices]

    y_sorted = y[sorted_indices]

    return auc(x_sorted, y_sorted)

def bootstrap_pr_auc_ci(y_true, y_probs, n_bootstraps=1000, alpha=0.05, seed=42):

    rng = np.random.RandomState(seed)

    bootstrapped_scores = []

    for _ in range(n_bootstraps):

        indices = rng.choice(len(y_probs), size=len(y_probs), replace=True)

        if len(np.unique(y_true[indices])) < 2:

            continue

        precision, recall, _ = precision_recall_curve(y_true[indices], y_probs[indices])

        score = safe_auc(recall, precision)

        bootstrapped_scores.append(score)

    sorted_scores = np.sort(bootstrapped_scores)

    ci_low = sorted_scores[int((alpha / 2) * len(sorted_scores))]

    ci_high = sorted_scores[int((1 - alpha / 2) * len(sorted_scores))]

    precision_, recall_, _ = precision_recall_curve(y_true, y_probs)

    auc_val = safe_auc(recall_, precision_)

    return ci_low, auc_val, ci_high

def plot_precision_recall_autogluon(model_path, model_name, test_csv_path, pr_csv_path=None, third_data_path=None,):

    n_bootstraps = 1000

    predictor = TabularPredictor.load(model_path)

    test_data = pd.read_csv(test_csv_path)

    label_column = predictor.label

    y_true = test_data[label_column].values

    X_test = test_data.drop(columns=[label_column])

    y_probs = predictor.predict_proba(X_test, model=model_name)[1].values

    precision, recall, thresholds = precision_recall_curve(y_true, y_probs)

    pr_auc = auc(recall, precision)

    f1_scores = 2 * (precision * recall) / (precision + recall + 1e-8)

    optimal_idx = np.argmax(f1_scores)

    optimal_threshold = thresholds[optimal_idx]

    optimal_f1 = f1_scores[optimal_idx]

    pr_auc_ci_low, pr_auc_val, pr_auc_ci_high = bootstrap_pr_auc_ci(y_true, y_probs, n_bootstraps=n_bootstraps)

    ag_label = (f'CatBoost_r128_BAG_L1 (AUC={pr_auc:.3f} [{pr_auc_ci_low:.3f}-{pr_auc_ci_high:.3f}])\n'

                f'Optimal Threshold={optimal_threshold:.3f} '

                f'F1={optimal_f1:.3f}')

    plt.figure(figsize=(10, 7))

    plt.plot(recall, precision, label=ag_label, color='blue')

    plt.scatter(recall[optimal_idx], precision[optimal_idx], color='red', marker='x', s=100)

    external_results = None

    if pr_csv_path:

        pr_df = pd.read_csv(pr_csv_path)

        p_ext = pr_df['Precision'].values

        r_ext = pr_df['Recall'].values

        t_ext = pr_df['Threshold'].values

        pr_auc_ext = auc(r_ext, p_ext)

        f1_ext = 2 * (p_ext * r_ext) / (p_ext + r_ext + 1e-8)

        idx_ext = np.argmax(f1_ext)

        thresh_ext = t_ext[idx_ext]

        optimal_f1_ext = f1_ext[idx_ext]

        df_test = pd.read_csv(test_csv_path)

        y_ext_true = df_test[predictor.label].values

        y_ext_probs = pr_df['Probability'].values if 'Probability' in pr_df.columns else predictor.predict_proba(X_test, model=model_name)[1].values

        pr_auc_ci_low_ext, pr_auc_val_ext, pr_auc_ci_high_ext = bootstrap_pr_auc_ci(y_ext_true, y_ext_probs, n_bootstraps=n_bootstraps, seed=456)

        ext_label = (f'Logistic Regression (AUC = {pr_auc_ext:.3f} [{pr_auc_ci_low_ext:.3f}-{pr_auc_ci_high_ext:.3f}])\n'

                     f'Optimal Threshold={thresh_ext:.3f} '

                     f'F1={optimal_f1_ext:.3f}')

        plt.plot(r_ext, p_ext, color='green', label=ext_label)

        plt.scatter(r_ext[idx_ext], p_ext[idx_ext], color='red', marker='x', s=100)

        external_results = {

            'pr_auc': pr_auc_ext,

            'pr_auc_ci': [pr_auc_ci_low_ext, pr_auc_ci_high_ext],

            'optimal_threshold': thresh_ext,

            'optimal_f1': optimal_f1_ext

        }

    third_model_results = None

    if third_data_path:

        third_df = pd.read_csv(third_data_path)

        third_labels = third_df['MACE'].values

        third_probs = third_df['prob'].values

        precision_third, recall_third, thresholds_third = precision_recall_curve(third_labels, third_probs)

        pr_auc_third = auc(recall_third, precision_third)

        f1_third = 2 * (precision_third * recall_third) / (precision_third + recall_third + 1e-8)

        idx_third = np.argmax(f1_third)

        optimal_threshold_third = thresholds_third[idx_third]

        optimal_f1_third = f1_third[idx_third]

        pr_auc_ci_low_third, pr_auc_val_third, pr_auc_ci_high_third = bootstrap_pr_auc_ci(

            third_labels, third_probs, n_bootstraps=n_bootstraps, seed=789

        )

        third_label = (

            f'Historical Model (AUC = {pr_auc_third:.3f} [{pr_auc_ci_low_third:.3f}-{pr_auc_ci_high_third:.3f}])\n'

            f'Optimal Threshold={optimal_threshold_third:.3f} '

            f'F1={optimal_f1_third:.3f}'

        )

        plt.plot(recall_third, precision_third, color='orange', label=third_label)

        plt.scatter(recall_third[idx_third], precision_third[idx_third], color='red', marker='x', s=100)

        third_model_results = {

            'pr_auc': pr_auc_third,

            'pr_auc_ci': [pr_auc_ci_low_third, pr_auc_ci_high_third],

            'optimal_threshold': optimal_threshold_third,

            'optimal_f1': optimal_f1_third

        }

    plt.xlabel('Recall')

    plt.ylabel('Precision')

    plt.title('Precision-Recall Curve Comparison')

    plt.legend()

    plt.grid(True)

    plt.tight_layout()

    plt.show()

    return {

        'autogluon': {

            'pr_auc': pr_auc,

            'pr_auc_ci': [pr_auc_ci_low, pr_auc_ci_high],

            'optimal_threshold': optimal_threshold,

            'optimal_f1': optimal_f1

        },

        'external_csv': external_results

    }
